# Risk adjustment performance of Charlson and Elixhauser comorbidities in ICD-9 and ICD-10 administrative databases

**DOI:** 10.1186/1472-6963-8-12

**Published:** 2008-01-14

**Authors:** Bing Li, Dewey Evans, Peter Faris, Stafford Dean, Hude Quan

**Affiliations:** 1 Department of Community Health Sciences, University of Calgary, Calgary, Alberta, Canada; 2 Quality, Safety and Health Information, Calgary Health Region, Calgary, Alberta, Canada; 3 British Columbia Cardiac Registry and Evaluation Services, Provincial Health Services Authority, Vancouver, British Columbia, Canada

## Abstract

**Background:**

The performance of the Charlson and Elixhauser comorbidity measures in predicting patient outcomes have been well validated with ICD-9 data but not with ICD-10 data, especially in disease specific patient cohorts. The objective of this study was to assess the performance of these two comorbidity measures in the prediction of in-hospital and 1 year mortality among patients with congestive heart failure (CHF), diabetes, chronic renal failure (CRF), stroke and patients undergoing coronary artery bypass grafting (CABG).

**Methods:**

A Canadian provincial hospital discharge administrative database was used to define 17 Charlson comorbidities and 30 Elixhauser comorbidities. C-statistic values were calculated to evaluate the performance of two measures. One year mortality information was obtained from the provincial Vital Statistics Department.

**Results:**

The absolute difference between ICD-9 and ICD-10 data in C-statistics ranged from 0 to 0.04 across five cohorts for the Charlson and Elixhauser comorbidity measures predicting in-hospital or 1 year mortality. In the models predicting in-hospital mortality using ICD-10 data, the C-statistics ranged from 0.62 (for stroke) – 0.82 (for diabetes) for Charlson measure and 0.62 (for stroke) to 0.83 (for CABG) for Elixhauser measure.

**Conclusion:**

The change in coding algorithms did not influence the performance of either the Charlson or Elixhauser comorbidity measures in the prediction of outcome. Both comorbidity measures were still valid prognostic indicators in the ICD-10 data and had a similar performance in predicting short and long term mortality in the ICD-9 and ICD-10 data.

## Background

Administrative hospital discharge abstract data are widely used in studies of healthcare outcomes. For valid and meaningful comparisons of providers, risk adjustment is essential since risk factors of outcomes are unevenly distributed across providers and variation in baseline status could make a major contribution to differences in patient outcomes. Risk adjustment is a complex construct that involves patient's socio-demographic factors (e.g. age, gender, and race), acute clinical stability, severity of primary disease, functional status, and burden of comorbidity [1]. As major determinants of patient outcomes, comorbidities or coexisting conditions have been studied extensively for decades. Many methods have been developed to measure and control comorbidities. The Charlson [2] and Elixhauser comorbidity measures [3] are two commonly used instruments for risk adjustment analyses.

Charlson *et al.*[2] studied numerous clinical conditions by reviewing inpatient hospital charts and assessing their relevance in the prediction of mortality. A weighted score was assigned to each of 17 comorbidities and the Charlson index was created as an indicator of disease burden. Elixhauser *et al.*[3] used sets of individual ICD-9-CM diagnosis codes to identify categories of comorbidities. They measured 30 individual comorbidities that are associated with mortality. The performance of the Charlson and Elixhauser measures in predicting poor outcomes has been assessed on various large populations [4-11]. These studies consistently demonstrated that they were valid prognostic measures of outcomes.

The World Health Organization adopted the first version of the International Classification of Disease (ICD) in 1900 to internationally monitor and compare mortality statistics and causes of death. Since then, the classification has been revised periodically to accommodate new knowledge of disease and health. The sixth revision, published in 1949, was more radical than the previous five revisions because this edition made it possible to record information from patient charts to compile morbidity statistics. Subsequent revisions were made in 1958 (7^th ^ed.), in 1968 (8^th ^ed.) and in 1979 (9^th ^ed.). The latest version ICD-10, was introduced in 1992 to replace the ICD-9 [12]. The United States modified ICD-9 by specifying many categories and extending coding rubrics to describe the clinical picture in more detail. These modifications resulted in the publication of ICD-9-CM in 1979 for coding diagnoses in patient charts [13].

Many countries such as Canada, Australia, New Zealand, Japan, China and European countries have already implemented the ICD-10. When compared to the ICD-9, the ICD-10 has a more comprehensive scope, effective structure, presentation and guidelines and allows for enhancements to accommodate newly discovered diseases [14]. The codes in ICD-10 are alphanumeric whereas codes in ICD-9 are numeric. Each code in ICD-10 starts with a letter (i.e. A to Z), followed by two numeric digits, a decimal point, and a digit (e.g. I21.4 for acute subendocardial myocardial infarction). In contrast, codes in ICD-9-CM begin with three digit numbers (i.e. 001 to 999), that are followed by a decimal and up to two digits (e.g. 410.7 for subendocardial infarction).

Since implementation of the ICD-10, researchers have been evaluating the performance of the Charlson and Elixhauser comorbidity measures using ICD-10 data. Quan *et al.*[7] developed ICD-10 coding algorithms to define Charlson and Elixhauser comorbidities and assessed the performance of the resulting algorithms in predicting in-hospital mortality. Sundarajan *et al.*[15] used Australian ICD-10 administrative data to evaluate the Charlson comorbidity measure in predicting in-hospital mortality. In both studies, the adaptation of the Charlson comorbidity measure for use with ICD-10 data yielded similar prevalence and prognosis information to a Charlson comorbidity measure based on ICD-9-CM.

These two studies assessed the performance of Charlson or Elixhauser measures in risk-adjustment in adult hospital admissions. However, these studies did not test the performance of the Charlson and Elixhauser comorbidity measures in predicting short and long term mortality in disease specific patient cohorts using ICD-10 administrative data. The present study addresses the above gap by using a large Canadian provincial hospital discharge administrative database containing ICD-9 and ICD-10 codes. Our study assesses the performance of Charlson and Elixhauser comorbidity measures in predicting in-hospital and one year mortality in five cohorts, including patients with congestive heart failure (CHF), diabetes, chronic renal failure (CRF), stroke and patients undergoing coronary artery bypass grafting (CABG).

## Methods

### Data source

This study employed hospital discharge abstract data (DAD) from the province of British Columbia, Canada between April 01, 1997 and March 31, 2004. The DAD contains demographic, administrative and clinical data for all hospital discharges in the province. British Columbia adopted the ICD-10 coding system in 2001. Prior to 2001, each discharge record contained up to 16 ICD-9 diagnosis codes and 10 procedure codes of the Canadian Classification of Diagnostic, Therapeutic and Surgical Procedures (CCP). Since 2001, each record contained up to 25 ICD-10 diagnosis codes and 20 procedure codes of the Canadian Classification of Health Interventions (CCI). For each diagnosis code in ICD-9 and ICD-10 data, a one digit 'diagnosis-type' code was assigned to specify the timing of diagnosis. Those occurring after hospital admission were assigned as complications. In order to be comparable with ICD-9 data, we truncated the ICD-10 data with the first 16 diagnosis codes and 10 CCI procedure codes.

### Study population

Five study populations were defined during 1997 and 2004 fiscal years (March 31 to April 1), four years prior to (1997 to 2000) and four years post (2001 to 2004) ICD-10 implementation. For each study cohort, we excluded non-British Columbia residents and those younger than 20 years of age. For stroke, diabetes, CHF, and CRF study cohorts, all admissions with diagnostic codes of each of these chronic conditions at the most responsible diagnosis coding field were selected (see Table 1 for ICD-9 and ICD-10 diagnostic codes). For patients with more than one admission, only the first admission was selected for each patient as the index admission. Conditions coded as a hospital complication were excluded through searching the 15 secondary diagnosis and diagnosis type coding fields. We further excluded those with length of hospital stay one day or less and discharged alive. The CABG study cohort was defined by including all hospitalizations with the 851 CCP code for ICD-9 and 1IJ76 CCI code for ICD-10 data. For those with multiple admissions, the latest admission was assigned to each patient as the index admission.

**Table 1:** Coding definition for four study cohorts

	*ICD-10*	*ICD-9*
**Congestive hear failure**	I43, I50, I099, I110, I130, I132, I255, I420, I425–I429, P290	4254, 4255, 4257, 4258, 4259, 428
**Diabetes**	E100, E101, E106, E108–E111, E116, E118–E121, E126, E128–E131, E136, E138–E141, E146, E148, E149, E102–E105, E107, E112–E115, E117, E122-E125, E127, E132–E135, E137, E142–E145, E147	250
**Chronic renal failure**	N18	585
**Stroke**	G45, H341, I60, I61, I63, I64	3623, 430, 431, 433–436

### Defining comorbidities and mortality

Quan *et al.*[7] developed the enhanced ICD-9-CM and ICD-10 coding algorithms to define Charlson and Elixhauser comorbidities. We slightly revised the enhanced ICD-9-CM coding algorithms by keeping the first 4 numerical digits for diagnosis codes with a 5th digit. Then, the slightly revised ICD-9-CM and original ICD-10 coding algorithms were employed respectively to define the 17 Charlson and 30 Elixhauser comorbidities in ICD-9 and ICD-10 data.

The outcome of interest was in-hospital and 1 year mortality. In-hospital mortality was defined using death flag in the DAD. To determine 1 year mortality after admission, inpatient records were linked with the British Columbia Vital Statistics registry using deterministic record linkage method. The common identifiers between two databases are personal health number, surname, sex, date of birth. The personal health number is unique numerical identifier but is not available in all death records. Therefore, we determined deaths based on matching on all of these identifiers. The possibility of linking one inpatient record to two deaths is very rare. We used the same matching strategy to match inpatients and death records in another Canadian province and found that the correct-linkage rate is about 98% [16]. The registry captures all deaths that occurred in the province. The mortality used in this study was all cause mortality.

### Statistical analysis

We first calculated the frequencies of Charlson and Elixhauser comorbidities in ICD-9 and ICD-10 data in each study cohort. Four logistic regression models were fit for ICD-9 data. Model 1 included in-hospital mortality as the dependent variable and age, sex, and Charlson comorbidities as the independent variable. Model 2 included in-hospital mortality as the dependent variable and age, sex, and Elixhauser comorbidities as the independent variables. Model 3 was fit using 1 year mortality after admission as the dependant variable and age, sex, and Charlson comorbidities as the independent variable. Model 4 included 1 year mortality after admission as the dependent variable and age, sex, and Elixhauser comorbidities as the independent variables. Then, we fit these four logistic regression models for ICD-10 data. In these eight models, we defined age and individual Charlson and Elixhauser comorbidities as binary variables. Age was categorized as < 65 and = 65 years old. Another option for adding Charlson comorbidities in the models is to weight each comorbidity and add the weighted index as an independent variable. A recent study [17] illustrated that the use of the individual comorbidities in model building performs better than the use of the weighted index for predicting mortality.

C-statistic values were calculated to assess performance of logistic regression models in discriminating mortality. The C-statistic is the area under the receiver operating characteristic (ROC) curve, measuring the ability of the predictive model to discriminate among those who do and do not die. A value of C = 0.5 indicates random prediction and C = 1 indicates perfect prediction [1]. We performed a bootstrapping procedure with 800 replications to determine approximate 95% confidence interval for C-statistic. Data analyses were conducted using SAS version 8.1 [18].

## Results

Frequencies of mortality and comorbidities in each study cohort are presented in Table 2. The mortality rates were quite similar for the chronic conditions of stroke, diabetes, CHF, and the procedure of CABG for the years spanned by the ICD-9 and ICD-10 databases. The mortality rate was however increased in the patients of CRF. There were also no obvious differences between two databases in the proportions of senior and female in each study cohort. When comparing the five cohorts, the diabetics were relatively younger than other four study cohorts. The portion of males in the CABG cohort was higher than in other cohorts, and accounted for almost 80% of the cohort. The frequencies of Charlson and Elixhauser comorbidities were generally similar for ICD-9 and ICD-10 data in each cohort. However renal failure was more frequent in ICD-9 than ICD-10 across the five study cohorts. Diabetes with complication was more prevalent in the ICD-10 data than in the ICD-9 data.

**Table 2:** Frequency of independent and dependent variables (%) in ICD-9 (1997–2000) and ICD-10 (2001–2004) data

	*Congestive Heart Failure*		*Diabetes*		*Chronic renal failure*		*Stroke*		*CABG*	
	
	*ICD-10* *(% of 25969)*	*ICD-9* *(% of 30008)*	*ICD-10* *(% of 11918)*	*ICD-9* *(% of 11411)*	*ICD-10* *(% of 1770)*	*ICD-9* *(% of 2076)*	*ICD-10* *(% of 22947)*	*ICD-9* *(% of 28984)*	*ICD-10* *(% of 10214)*	*ICD-9* *(% of 9578)*
In-hospital Mortality	12.4	11.6	4.2	3.7	16.8	13.2	17.0	15.4	2.9	3.3
One year Mortality	40.1	39.7	19.0	17.7	42.0	34.1	29.2	26.1	5.1	5.5
Male	50.2	51.5	55.9	54.4	55.0	56.7	49.6	50.9	79.7	78.6
Senior	85.9	86.8	45.3	44.8	65.0	58.0	77.9	78.5	60.4	59.9
Charlson comorbidities										
Myocardial infarction	9.6	15.1	3.6	4.9	3.6	5.7	3.4	5.2	19.3	36.1
Congestive heart failure	-	-	8.1	8.2	18.9	15.1	4.7	4.3	19.6	16.0
Peripheral vascular disease	2.1	4.9	11.2	10.2	3.6	3.3	1.7	3.8	5.2	7.4
Cerebrovascular disease	2.2	3.6	2.5	3.8	2.2	3.0	-	-	5.4	6.8
Dementia	3.8	2.9	3.4	2.4	3.2	1.0	5.6	3.9	0.5	0.1
Chronic pulmonary disease	16.2	19.6	3.5	5.0	5.5	6.1	3.8	5.2	4.7	8.0
Rheumatic disease	0.8	1.4	0.7	0.8	2.0	2.5	0.6	0.9	0.3	0.6
Peptic ulcer disease	0.4	0.3	0.4	0.3	0.3	0.6	0.3	0.3	0.8	1.2
Mild liver disease	0.8	1.0	1.2	1.6	1.7	1.5	0.3	0.4	0.2	0.5
Diabetes without chronic complication	24.0	23.1	-	-	16.3	18.3	17.1	15.5	25.6	22.8
Diabetes with chronic complication	4.2	1.8	-	-	6.4	2.7	1.5	0.8	5.3	2.8
Hemiplegia or paraplegia	0.4	0.6	0.6	1.1	0.5	0.6	14.0	16.1	0.7	0.9
Renal disease	14.2	10.2	17.2	14.9	-	-	2.6	1.6	5.9	3.1
Any malignancy	2.4	2.7	2.0	1.7	4.7	3.1	2.1	2.0	0.9	1.0
Moderate or severe liver disease	0.4	0.3	0.3	0.3	0.7	0.4	0.1	0.1	0.1	0.1
Metastatic solid tumor	0.7	0.7	0.8	0.5	1.6	0.5	0.8	0.8	0.1	0.0
AIDS/HIV	0.0	0.1	0.1	0.1	0.0	0.0	0.0	0.1	0.0	0.1
Elixhauser comorbidities										
Congestive heart failure	-	-	8.1	8.2	18.9	15.1	4.7	4.3	19.6	16.0
Cardiac arrhythmia	28.2	30.0	4.8	6.0	8.5	7.4	13.5	13.8	31.7	36.3
Valvular disease	10.5	12.4	0.8	1.3	2.8	2.6	1.5	2.1	20.3	17.4
Pulmonary circulation disorders	1.9	2.0	0.3	0.2	0.5	0.5	0.4	0.3	1.3	1.9
Peripheral vascular disorders	2.1	4.9	11.2	10.2	3.6	3.3	1.7	3.8	5.2	7.4
Hypertension	19.4	20.2	20.0	22.2	23.8	22.3	31.4	32.4	33.6	41.6
Hypertension, uncomplicated	15.9	18.1	14.4	17.1	14.5	19.7	30.2	31.7	30.7	39.9
Hypertension, complicated	3.5	2.1	5.6	5.1	9.3	2.6	1.2	0.7	2.9	1.7
Paralysis	0.4	0.6	0.6	1.1	0.5	0.6	14.0	16.1	0.7	0.9
Other neurological disorders	1.1	1.5	2.2	2.3	2.2	2.6	8.2	8.1	1.0	1.4
Chronic pulmonary disease	16.2	19.6	3.5	5.0	5.5	6.1	3.8	5.2	4.7	8.0
Diabetes, uncomplicated	23.3	22.9	-	-	15.7	18.1	16.8	15.3	25.4	22.7
Diabetes, complicated	4.8	2.0	-	-	7.0	2.8	1.7	1.0	5.5	2.9
Hypothyroidism	1.6	2.4	1.6	2.0	1.1	1.5	1.3	1.4	0.8	1.3
Renal failure	14.2	8.7	17.2	10.4	-	-	2.6	1.1	5.9	1.6
Liver disease	1.1	1.2	1.4	1.7	2.2	1.8	0.4	0.4	0.3	0.5
Peptic ulcer disease excluding bleeding	0.2	0.2	0.3	0.2	0.2	0.2	0.1	0.1	0.3	0.6
AIDS/HIV	0.0	0.1	0.1	0.1	0.0	0.0	0.0	0.1	0.0	0.1
Lymphoma	0.5	0.5	0.3	0.3	1.4	0.9	0.2	0.2	0.1	0.1
Metastatic cancer	0.7	0.7	0.8	0.5	1.6	0.5	0.8	0.8	0.1	0.0
Solid tumor without metastasis	1.6	1.9	1.5	1.2	3.2	2.0	1.8	1.6	0.6	0.7
Rheumatoid arthritis/collagen vascular diseases	0.9	1.6	0.7	0.9	2.7	4.2	0.6	1.0	0.5	0.7
Coagulopathy	0.7	0.5	0.4	0.4	1.6	0.9	0.7	0.5	1.7	1.5
Obesity	1.4	1.5	1.8	2.5	0.9	0.8	0.4	0.5	1.3	2.6
Weight loss	0.6	0.6	1.0	0.9	2.1	1.5	0.4	0.3	0.2	0.3
Fluid and electrolyte disorders	4.7	4.0	8.4	8.7	12.3	9.9	2.4	2.0	0.9	1.1
Blood loss anaemia	0.9	1.4	0.4	0.8	1.1	1.7	0.2	0.3	0.1	0.2
Deficiency anaemia	2.0	0.6	1.4	0.5	2.4	0.7	0.6	0.2	0.4	0.1
Alcohol abuse	1.7	2.0	3.3	3.0	1.4	1.0	1.8	1.2	1.0	0.8
Drug abuse	0.3	0.3	1.9	2.0	0.8	0.2	0.4	0.2	0.2	0.3
Psychoses	0.3	1.4	0.8	2.0	0.8	2.1	0.3	1.0	2.6	0.5
Depression	1.7	1.9	3.5	3.3	2.7	2.4	2.4	1.8	1.2	1.2

Tables 3 and 4 present the C-statistics of Charlson and Elixhauser comorbidity measures in predicting mortality in the ICD-9 and ICD-10 data. The C-statistics were similar between ICD-9 and ICD-10 datasets for each study cohort using either the Charlson or Elixhauser comorbidity measure to predict in-hospital or 1 year mortality. The absolute difference in C-statistics across cohorts ranged from 0 to 0.04. Using Charlson comorbidities to predict in-hospital mortality, the C-statistics for five cohorts ranged from 0.62 (for stroke) to 0.82 (for diabetes) in the ICD-10 data. Using Elixhauser measure to predict in-hospital mortality, the C-statistics ranged from 0.62 (for stroke) to 0.83 (for CABG) in the ICD-10 data. The C-statistics increased when these two measures predicted 1 year mortality in stroke cohort but declined for remaining 4 cohorts studied.

**Table 3:** Performance of Charlson and Elixhauser comorbidities in predicting in-hospital mortality (C-statistic, 95% confidence interval)

	Congestive heart failure		Diabetes		Chronic renal failure		Stroke		CABG	
	
	*ICD-10*	*ICD-9*	*ICD-10*	*ICD-9*	*ICD-10*	*ICD-9*	*ICD-10*	*ICD-9*	*ICD-10*	*ICD-9*
Charlson	0.65	0.66	0.82	0.83	0.74	0.76	0.62	0.65	0.80	0.77
comorbidities	(0.65–0.66)	(0.65–0.66)	(0.81–0.83)	(0.81–0.84)	(0.72–0.76)	(0.75–0.78)	(0.61–0.62)	(0.64–0.65)	(0.77–0.82)	(0.76–0.80)
Elixhauser	0.66	0.66	0.82	0.83	0.75	0.78	0.62	0.66	0.83	0.82
comorbidities	(0.65–0.67)	(0.65–0.66)	(0.81–0.83)	(0.81–0.84)	(0.74–0.78)	(0.76–0.80)	(0.62–0.63)	(0.65–0.66)	(0.82–0.85)	(0.81–0.84)

**Table 4:** Performance of Charlson and Elixhauser comorbidities in predicting one year mortality (C-statistic, 95% confidence interval)

	Congestive heart failure		Diabetes		Chronic renal failure		Stroke		CABG	
	
	*ICD-10*	*ICD-9*	*ICD-10*	*ICD-9*	*ICD-10*	*ICD-9*	*ICD-10*	*ICD-9*	*ICD-10*	*ICD-9*
Charlson	0.63	0.64	0.77	0.76	0.76	0.76	0.65	0.66	0.77	0.73
comorbidities	(0.63–0.63)	(0.63–0.64)	(0.76–0.77)	(0.76–0.77)	(0.75–0.78)	(0.75–0.78)	(0.65–0.65)	(0.66–0.66)	(0.76–0.79)	(0.72–0.77)
Elixhauser	0.64	0.64	0.77	0.76	0.78	0.77	0.65	0.67	0.78	0.79
comorbidities	(0.64–0.65)	(0.64–0.65)	(0.77–0.78)	(0.76–0.77)	(0.77–0.79)	(0.76–0.79)	(0.65–0.66)	(0.67–0.67)	(0.75–0.81)	(0.76–0.80)

## Discussion

We compared the performance of the Charlson and the Elixhauser comorbidity measures in predicting short and long term mortality among patients with CHF, diabetes, CRF, stroke, and CABG between ICD-9 and ICD-10 Canadian hospital discharge abstract databases. We found that most comorbidities studied had similar frequencies across ICD-9 and ICD-10 databases although variations were observed for some comorbidities. The performance of the Charlson and Elixhauser comorbidity measures in discriminating mortality was similar in ICD-9 data as well as ICD-10 data across five study cohorts.

In the patients with CHF and stroke, the C-statistic values for the predicting in-hospital or 1 year mortality were notably low with values of 0.63 and 0.66 for CHF and 0.62 and 0.67 for stroke, respectively. The low values might reflect the fact that some powerful clinical predictors of poor outcomes were not included in these measures due to a lack of information in the administrative databases, such as ejection fraction and severity of stroke. We also demonstrated that both of the comorbidity measures performed better in predicting 1-year mortality than in-hospital mortality in the patients with stroke. This finding provides evidence that severity and type of stroke (such as hemorrhage or ischemic attach) should be considered to improve risk adjustment performance. In contrast to stroke, the performance of the Charlson and Elixhauser measures in discriminating mortality among patients with CHF, diabetes and undergoing CABG declined with length of prediction.

The quality of data used for risk adjustment will affect the reliability and validity of the model. The introduction of new coding schemata, such as the ICD-10, presents significant issues regarding coding accuracy and completeness of clinical information. The magnitude of changes between coding schemes from ICD-9 to ICD-10 may induce coding errors unless efforts are taken to prepare coders for the changes through education and practice sessions and post implementation monitoring and quality improvement steps to reduce any notable errors or misinterpretations in coding practices. Our findings showed that most comorbidities had similar frequencies across ICD-9 and ICD-10 databases and the performance of two co-morbidity measures in discriminating mortality was similar between ICD-9 data and ICD-10 datasets across five study cohorts. The results suggest that the implementation of ICD-9 and ICD-10 might have similar validity. These results may reflect efforts to improve the quality of coding pre and post implementation of ICD-10 by the British Columbia Ministry of Health.

ICD-10 was implemented in Canadian hospitals in different years for each province. British Columbia introduced ICD-10 in 2001 fiscal year. Before the implementation, detailed coding standards for the new classification system were developed, and health records professionals were intensively trained. Following the collection of the first year's data, data quality concerns in the ICD-10 data were collected and reviewed. The review led to various initiatives to further improve training and data quality, including clarification of coding requirements for certain conditions, and re-abstracting records for validity assessment. The first post implementation pan-Canadian re-abstraction study was taken in 2003/04 [19]. At the same time, a Data Quality Framework was introduced by CIHI and new edit checks were implemented to address incorrect application of coding standards and other data quality issues. An ongoing schedule for national re-abstraction studies has been established.

Limitations of our study have to be acknowledged before making conclusions. We assessed comorbidity measures in two different periods, 1997–2000 for ICD-9 and 2001–2004 for ICD-10. We could not exclude contribution of temporal change in disease prevalence and data quality to model performances. Both Charlson and Elixhauser measures consist of chronic diseases and their prevalence may not vary much in such a short period as our findings of similar frequencies for many comorbidities across two databases. The second limitation is that our findings might not be applicable to other diseases or procedures. We selected inpatients with four conditions and one procedure. We found the performance of Charlson and Elixhauser comorbidity measures varied across five study cohorts. Therefore one should be cautious when generalizing our findings beyond these conditions and procedure. The third limitation is that we defined comorbidities using index admission. Employing previous admission records to define comorbidities will increase prevalence of these conditions, resulting in a better risk adjustment [20,21]. However, our study conclusions may not be altered due to the length of the lookback because the same method of ascertaining comorbidities was applied to both databases.

## Conclusion

In conclusion, the change in disease coding system from ICD-9 to ICD-10 did not impact the performance of Charlson and Elixhauser comorbidity measures in the prediction of mortality. Both measures were acceptable prognostic predictors for diabetes, CRF, and CABG in outcome research and performed similarly.

## Competing interests

The author(s) declare that they have no competing interests.

## Authors' contributions

BL contributed to the study design, statistical analysis, interpretation and writing of the manuscript. DE contributed to the interpretation, and writing of the manuscript. PF contributed to the interpretation and writing of the manuscript. SD contributed to the interpretation and writing of the manuscript. HQ contributed to the interpretation and writing of the manuscript. All authors have read and approved the final version of the manuscript.

## Pre-publication history

The pre-publication history for this paper can be accessed here:


